# Current-induced zero-field domain wall depinning in cylindrical nanowires

**DOI:** 10.1038/s41598-022-22623-0

**Published:** 2022-11-14

**Authors:** Julian A. Moreno, Jurgen Kosel

**Affiliations:** 1 King Abdullah University of Science and Technology, Thuwal, 23955 Saudi Arabia; 2 Silicon Austria Labs, 9524 Villach, Austria

**Keywords:** Magnetic devices, Nanoscale materials, Electronic and spintronic devices

## Abstract

Multi-segmented cylindrical nanowires have properties that make them attractive for high-density, high-speed logic and memory applications. Investigations of the current-induced domain wall motion in cylindrical nanowires have, so far, typically been conducted with a background magnetic field. However, if performed at zero external field, they would be much more viable for their use in prospective electronic devices. Here, we present an all-magneto electrical method to consistently pin domain walls in multi-segmented nanowires and induce their de-pinning using current pulses. The experiments were conducted with compositionally modulated three-segmented nickel/cobalt/nickel and two-segmented cobalt/nickel nanowires of 190 and 150 nm diameter, respectively, where the soft/hard magnetic texture has been fairly studied. We find that for the 3 segmented nanowire, the domain wall can be de-pinned independent of the polarity of the pulse, while for the 2 segmented nanowire the domain wall de-pins only for one polarity. Applying current pulses of 1 × 10^12^ A/m^2^, we use a pulse width of 22 ns to estimate a lower boundary for the domain wall speed of 634.54 m/s in cobalt. We study the resistive heating effect from the DC measurement current to find a temperature increase of no more than 2 °C after more than 20 h of tests.

## Introduction

Over the past decade great advances have been made in the engineering of domain wall (DW) pinning sites within cylindrical nanowires (NWs) using length^1^, diameter^2^ or compositional modulations^3^. These engineered pinning sites are essential for a controlled study of the DWs’ magnetic texture and its interaction with electrical currents, which may enable future storage devices^4^. Although some experimental studies exist on the pinned DW’s texture^3,5,6^, there are not many reports regarding current-induced DW de-pinning in cylindrical NWs^7,8^ with only this last reference, to the best of our knowledge, reporting DW de-pinning from defects at zero field. Some of the reasons for this lack of current-induced DW de-pinning studies are the technical difficulties of electrically contacting single NWs with DW pinning sites in-between the electrodes and the tracking of the DW, once it has been de-pinned by a current pulse.

Perhaps one of the most experimentally studied system for engineering DW pinning sites is the magnetically soft/hard compositionally modulated NW. A good representative of such system, which allows the control of crystal and shape anisotropy, is the Co/Ni modulated NW, whose DW-pinning capabilities have been explored both in arrays^9,10^ as well as individually^3^. In this system, for long enough NWs, a DW is known to pin inside the Ni segment close to the Co/Ni interface^3,11^. Such DW represents a magnetization gradient along the length of the NW, which is needed for conduction electrons whose spins do not follow the direction of the local moment, to exert a spin torque on the magnetization, ultimately moving the DW^12^.

In this work, we present an all-magnetoelectrical method to pin a DW in NWs with engineered pinning sites and apply current pulses to such pinned DWs at zero applied external field, in order to move (de-pin) it. Similar to other methods, which use MFM^8^ or TEM^7^, this is not a real-time measurement and the effect of the current pulse on the DW is only known seconds or minutes later, after performing an additional probing measurement.

## Results

### Magnetic states

**Figure 1:**
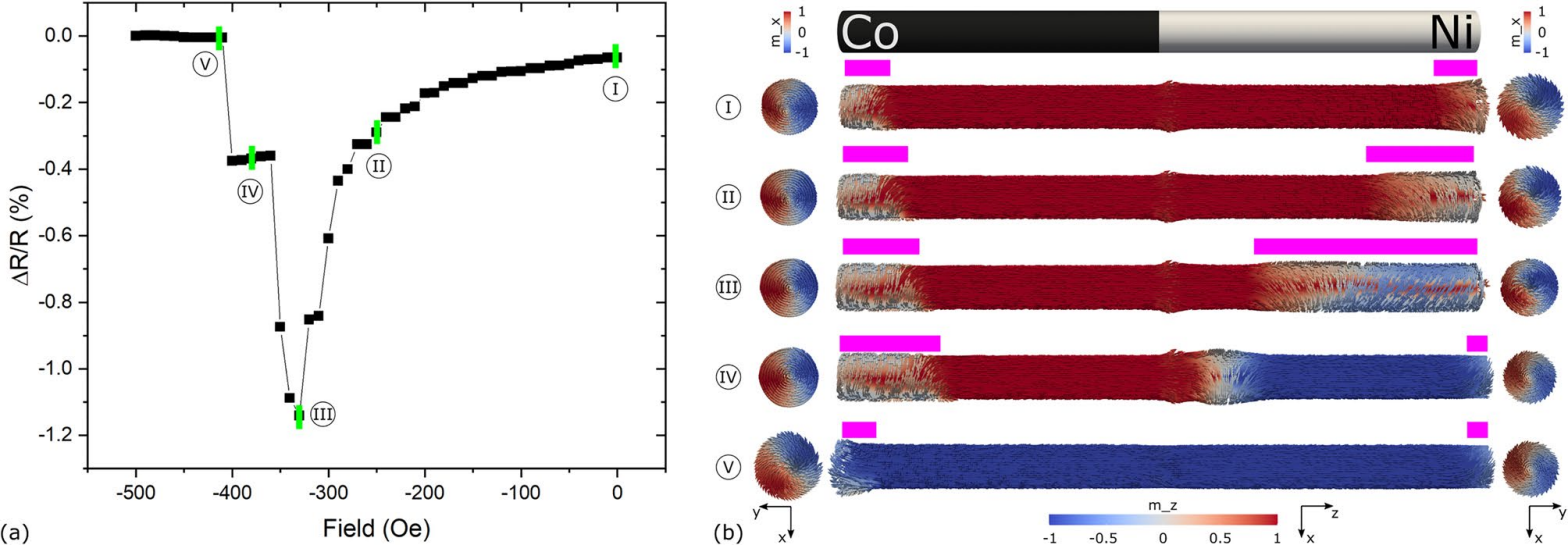
(**a**) Simulated AMR curve of a Co/Ni NW starting from remanence and increasing the field towards the negative direction. Representative magnetization states I to V at certain field values are labeled by green bars. (**b**) Side views composed of cross-sections and sparse glyph representations of the magnetization states I to V in (**a**) in addition to top views next to their respective ends. Side views are colored by m_z while top views are colored by m_x (see axes directions at the bottom).

The presence of a DW or a non-axial magnetic configuration along the NW was measured using the anisotropic magnetoresistance (AMR) effect. The AMR depends on the direction of the measurement current with respect to the one of the magnetization^13^. Therefore, simplifying, it gives an overall measure of the portion of the NW’s magnetization M that is not parallel to the direction of the applied measurement current I_m_. At ± 3 kOe (and remanence due to high shape anisotropy), most of the NW’s magnetization is in the same direction as the measurement current, i.e., along the NW’s long axis, resulting in saturated high resistance states, such as states I and V in Fig. 1. This figure shows a simulated AMR curve of a Co/Ni NW starting from remanence (state I) and increasing the field towards the negative direction until the NW’s magnetization is reversed (state V). Side views consisting on cross sections and sparse glyph representations of the selected magnetization states I to V in Fig. 1a are shown in Fig. 1b with the Co on the left and Ni on the right. Starting at remanence (state I), as the field increases, magnetization vortices appear at the ends of the NW, driving magnetization away from the axial direction and resulting in lower resistance states, as is shown in states II and III in Fig. 1 with a qualitative measure of the vortices indicated with pink bars above the NW side views in Fig. 1b.

These vortices minimize the magnetostatic energy. As the external applied field increases, the vortices grow larger in Ni, since its saturation magnetization, exchange energy and magneto crystalline anisotropy are at least one order of magnitude less than the ones of Co, i.e., it is a “softer” magnetic material. Notice also that this happens regardless of both having the same shape anisotropy (same geometry). When the vortices reach a critical “size” at a certain field, as shown in state III of Fig. 1, a DW nucleates and propagates from the right (Ni) end and is pinned at some point within the Ni segment close to the interface^14^, increasing the axial component of the magnetization as indicated by state IV (a detailed view of the DW can be found in supporting Fig. S1). Finally, when the field is further increased, the DW de-pins and propagates to the opposite (Co) end, leaving most of the magnetization in the axial direction again, as indicated by state V in Fig. 1. Note that NWs with vortices at their ends and pinned DWs within their length have a lower resistance, compared to uniformly-magnetized NWs, as they contain magnetization textures out of axis.

For the experimental study, the system had to first be brought to state IV of Fig. 1. This was achieved by saturating the magnetization by applying + 3 kOe, decreasing the field, inverting it and increasing it only until the first resistance jump was reached. Once in this state, the magnetic field was ramped down to zero, where two scenarios could occur: either the magnetization reversed to state I, i.e., the DW “returned” to where it was nucleated from, or the DW remained pinned in state IV of Fig. 1 at zero field. After returning the field to zero from state IV, a way to test for the outcome of these scenarios was to ramp the field up again in the negative direction. If the magnetization returned to state I, two resistance jumps would be observed in the AMR curve: one related to the transition from state III to state IV and one related to the transition from state IV to state V. On the other hand, if the DW remained pinned, i.e., state IV was conserved at zero field, once the field ramps up again, only the resistance jump related to the transition from state IV to V would be observed. As will be seen from all measurements in the following sections and in supporting Figs. S3, S4, after being pinned (state IV), the DW never returned to state III after reducing the field to zero, i.e., only one resistance jump was always observed when ramping the field up again. In addition to this, if the DW remained pinned at zero field and a current pulse was used to induce its de-pinning, i.e., take the magnetization to state V, no resistance jump would be observed once the field was ramped up again.

### Nanowire composition

**Figure 2:**
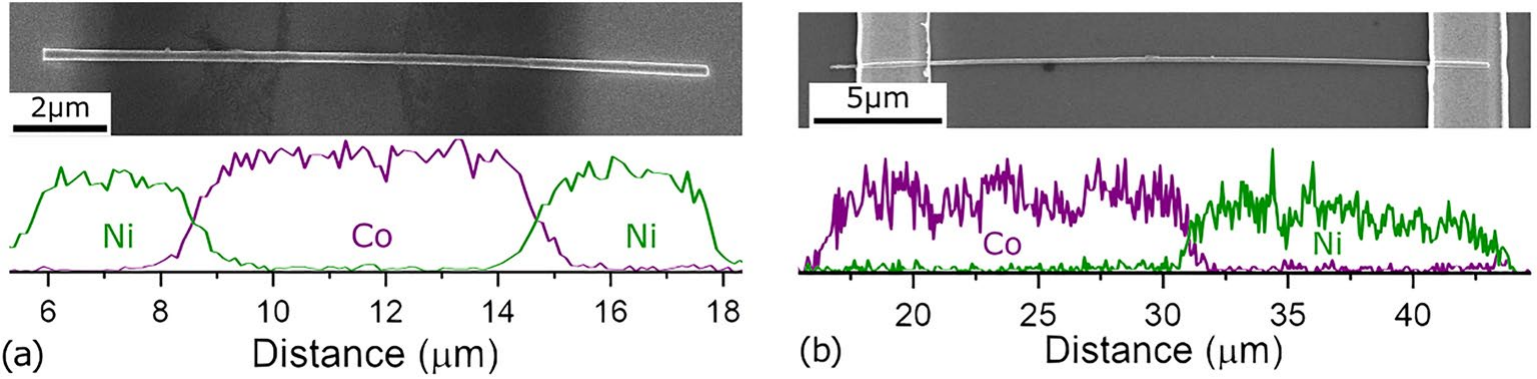
Scanning electron microscope image (top) and composition spectra (bottom) of (**a**) Ni/Co/Ni and (**b**) Co/Ni nanowires.

The nanowire composition was first investigated using energy-dispersive X-ray (EDX) spectroscopy within a scanning electron microscope (SEM), while they were inside the membrane to examine their total length and the lengths of the segments. These spectra were then compared to the lengths of the released, contacted NWs to infer their segments length. Figure 2 shows the SEM images of the contacted (a) Ni/Co/Ni and (b) Co/Ni NWs (top), as well as their respective EDX spectra (bottom).Measuring the portions at the positions where the Co and Ni spectra cross, we found the lengths of the segments of Ni/Co/Ni as [3.37/7.31/3.64] µm (Fig. 2a) and of Co/Ni as [13.96/11.71] µm (Fig. 2b). The average diameters were found to be 188 nm and 148 nm for the three- and two-segmented NWs in Fig. 2a,b, respectively. These values were used to calculate the current densities applied both for the continuous AMR measurements and pulse amplitudes.

### Three-segmented nanowire

**Figure 3:**
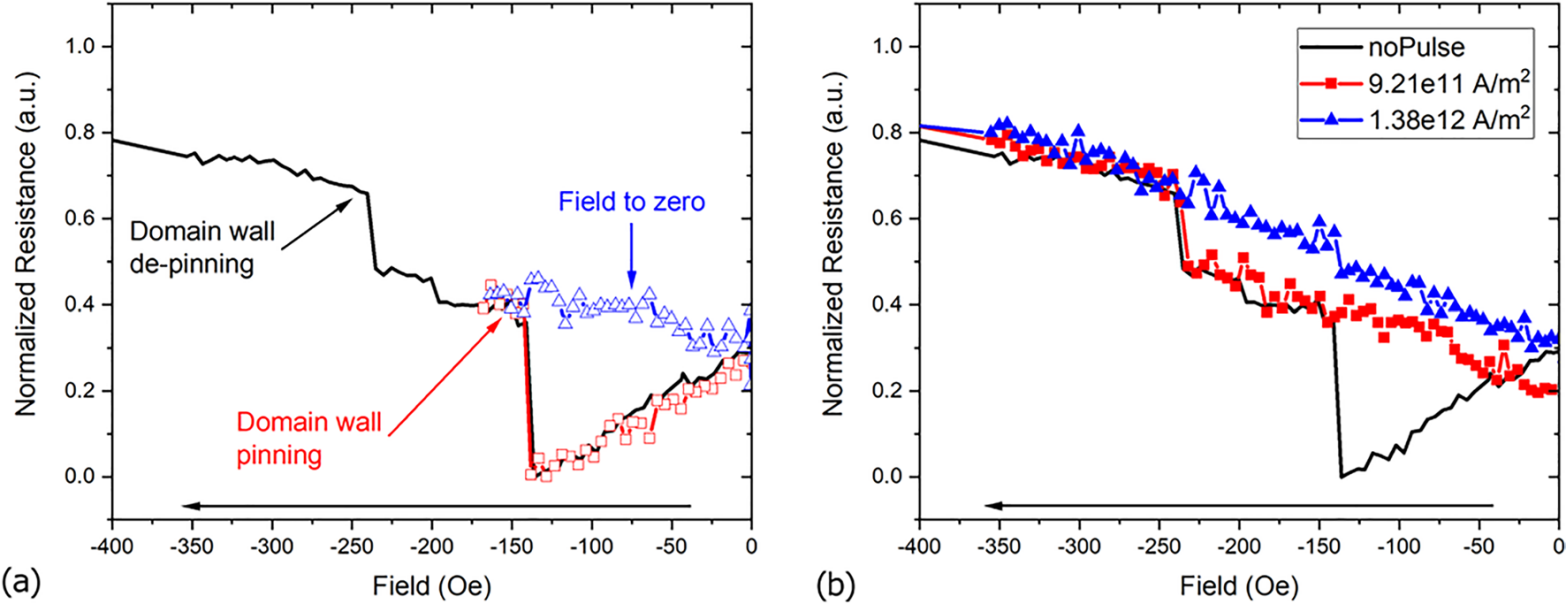
(**a**) Anisotropic magneto-resistance measurement of Ni/Co/Ni cylindrical nanowire with a pinned domain wall and the subsequent reduction of the external field to zero, after which nanosecond square current pulses were applied. (**b**) Magnetoresistance profile after two different current pulses were applied at zero field: 9.21 × 10^11^ A/m^2^, 10 ns width (red squares), the de-pinning resistance jump indicates the domain wall was not de-pinned; 1.38 × 10^12^ A/m^2^, 20 ns (blue triangles) results in the de-pinning of the DW as the depinning resistance jump is not observed.

Current pulses at zero field were first tested in the Ni/Co/Ni NW, shown in Fig. 2a, where previous experiments showed that, compared to two-segmented NWs, state IV persisted for higher negative fields before switching to state V as the field was ramped up (the “plateau” was wider)^15^. Furthermore, the pinning event (state IV) was more evident in the resistance value (discernible from states III and V) of the AMR curve compared to two-segmented NWs. The normalized AMR values from such NW, with its long axis parallel to the applied field, and in a range relevant to the pinning/de-pinning events is shown in Fig. 3 (solid line). This curve starts from a saturated magnetization state in the “right” direction (+ 3 kOe, not shown) and proceeds to a saturated state in the “left” direction (− 3 kOe, not shown), as indicated by the arrow at the bottom (for a full curve, see Supplementary Fig. S2).

Using the method described before, the NW was brought to a magnetic state in which a DW was pinned, as seen in Fig. 3a, red open squares (equivalent to state IV in Fig. 1). After this, the field was reduced to 0 ± 5 Oe (open blue triangles in Fig. 3a) where current pulses of different magnitudes were applied before ramping the field up again to find out about the resulting magnetization state. The outcome of two representative pulses is displayed in Fig. 3b: after a current pulse of 9.21 × 10^11^ A/m^2^ and 10 ns width was applied (solid red squares), one resistance jump was observed during the field ramp up, meaning that the applied pulse did not induce the de-pinning of the DW. On the other hand, after a current pulse of 1.38 × 10^12^ A/m^2^ and 20 ns width was used, neither of the resistance jumps were observed, meaning the magnetization assumed a state equivalent to state V in Fig. 1, i.e., the current pulse induced the de-pinning of the DW, reversing the magnetization.

**Figure 4:**
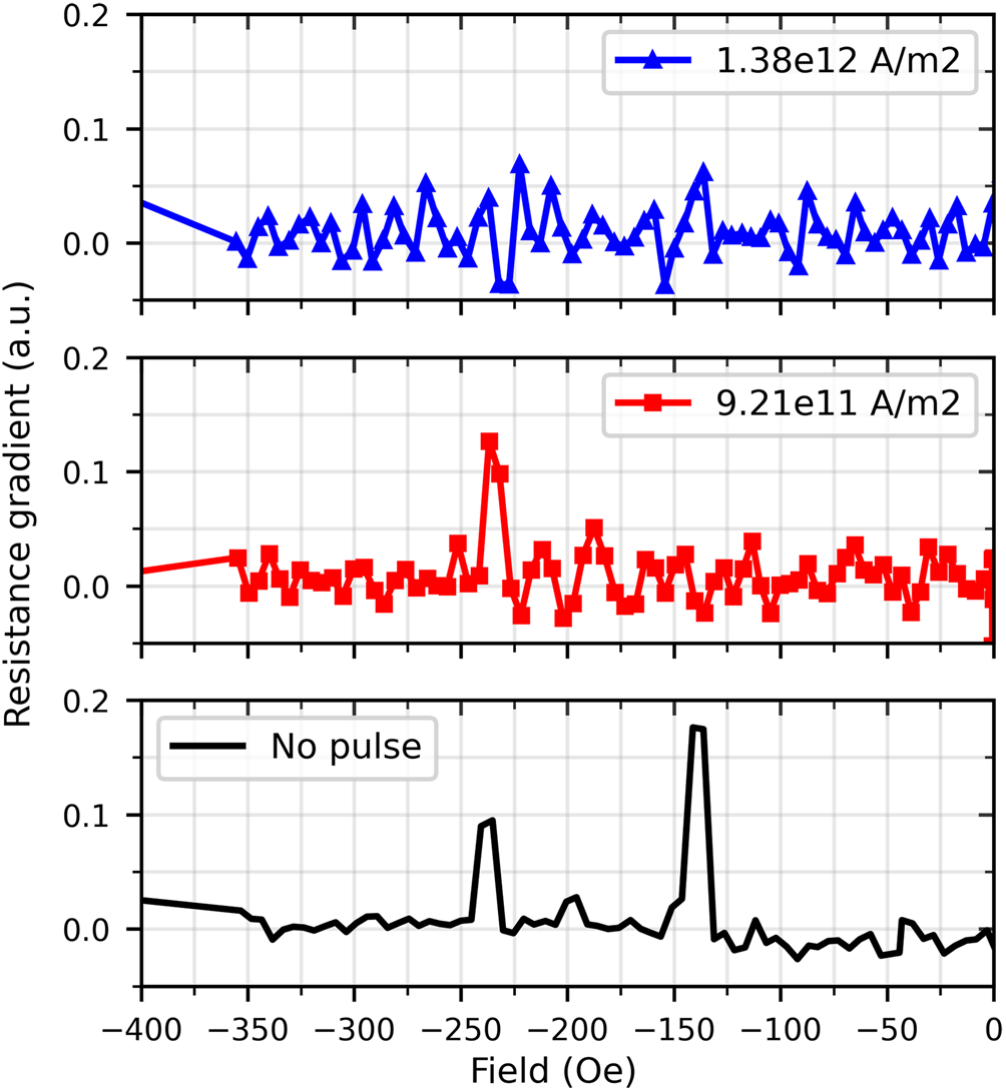
Numerical gradients of the resistances obtained from the curves in Fig. 3b.

One way to quantify the effect of the current pulses is to plot the gradient of the AMR curves in Fig. 3b against the applied field and identify the peaks at the fields at which the resistance jumps occurred, as shown in Fig. 4. From Fig. 4**,** when no pulse was applied, two peaks at approx. − 140 and − 240 Oe are displayed, corresponding to the resistance jumps of the black solid line in Fig. 3b. When the current pulse did not induce the de-pinning of the DW (solid red squares in Fig. 4), only the peak around − 240 Oe can be observed, corresponding to the de-pinning resistance jump in Fig. 3a**.** On the other hand, when the current pulse induced the de-pinning of the DW (solid blue triangles in Fig. 4), no distinct peak can be seen over the noise. For this three-segment NW, all measurements can be found in the Supplementary Fig. S3.

In the three-segmented NW presented, it is possible for two DWs to be pinned near the two Ni/Co interfaces at the same time although, realistically, due to imperfections such as different geometries at the NW ends^16^, one DW would nucleate. This makes it difficult to track which segments are being switched, i.e., which DW is moving and in which direction as both may be moving and collapsing into each other at the same time when the de-pinning jump is observed. This difficulty in tracking the DW gets more complex as the number of segments increases because the AMR value of multiple-pinned DWs does not vary greatly, when only one DW de-pins^15^.

### Two-segmented nanowire

**Figure 5:**
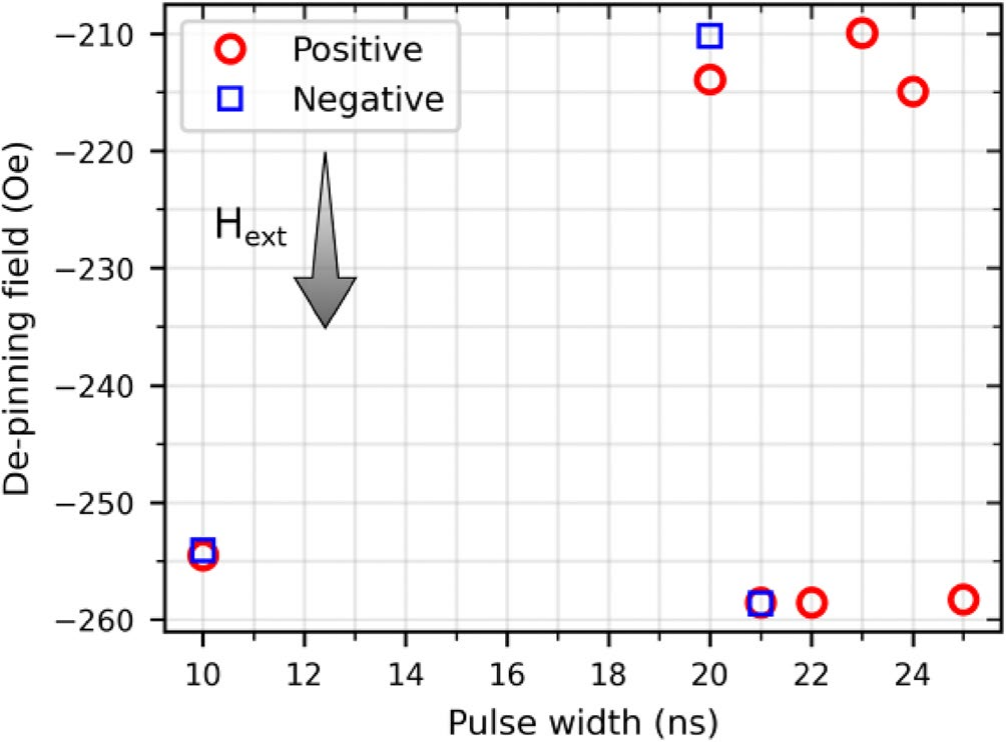
De-pinning field observed after applying pulses of 1 × 10^12^ A/m^2^ and different pulse widths and polarities. For pulse widths longer than 22 ns, negative pulses induce the de-pinning of the DW and no de-pinning field is observed.

In order to track a single DW, the described method was applied to the two-segmented Co/Ni NW, shown in Fig. 2b**,** where only one DW can be pinned, and whose magnetization states correspond to the ones presented in Fig. 1. The experiment was setup in a way that for a “Positive” current pulse, electrons flowed from left to right (from Co to Ni) while for a “Negative” current pulse, electrons flowed from right to left (from Ni to Co). Figure 5 shows the observed fields at which a peak appeared in the gradient of the AMR curves, after current pulses of 1 × 10^12^ A/m^2^ with different pulse widths and polarities were applied. The appearance of the peaks means the current pulses did not induce the de-pinning of the DW (all measurements can be found in the Supplementary Fig. S4). As seen in Fig. 5 (and Supplementary Fig. S4) for pulses of 22 ns and longer, domain wall de-pinning was observed for positive but not for negative current pulses, i.e., the de-pinning event was dependent on the direction of the applied current pulse and only happened when electrons flowed from Ni to Co.

In addition to this, Fig. 5 also shows that the de-pinning field value differs by around 40 Oe for some measurements (from around − 215 to − 260 Oe). This difference was also found in measurements performed without applying current pulses in two and three-segmented NWs and can be found in the Supplementary Fig. S5a,b for two and three-segmented NWs, respectively.

### Current-induced heating

In addition to the current pulses, as the NW was constantly under an applied measurement current of 2 × 10^8^ A/m^2^, to measure the AMR, heating was expected to emerge to some extent. For every measurement in which a pulse was applied, a measurement without a pulse was first conducted in order to check for any changes in the DW pinning field and as an additional reset of the NW (saturating to + 3 kOe in every measurement was considered as a reset). AMR measurements took 30 min each, when no pulse was applied and 39 min when pulses were applied (additional time was needed to return the field to zero and ramp it back up again after the pulse was applied). Overall, the two-segmented Co/Ni NW was under constant applied current for a total of 20.7 h.

The last day of measurements is summarized in Fig. S6, where the minimum resistance, extracted from all reported AMR curves, is shown. It is seen that each subsequent measurement increases the minimum resistance and that the system does not completely cool down in the time needed to input new parameters for the next experiment (some seconds to minutes). The reset to minimum resistance only happened when the system was allowed to cool down overnight.

## Discussion

A method was presented to consistently pin DWs at engineered locations within a two and three-segmented NW at zero field and induce their de-pinning using current pulses.

For the two-segmented NW, it was found that the DW de-pinning was dependent on the applied current pulse direction and it only occurred when electrons flowed from the Ni into the Co. This asymmetry could arise from the higher out-of-axis crystal anisotropy of Co, compared to the almost uniaxial in-axis crystal anisotropy of Ni^17^. When electrons flow from Co to Ni, they interact with the DW with an out-of-axis spin component, which interacts with the axial component of the DW; while when electrons flow from Ni to Co, they interact with the DW with a higher in-axis spin component. As the DW was de-pinned when using pulses with widths of 22 ns and higher, a minimum DW speed of 634.54 m/s could be estimated assuming it moved over the Co’s segment length (13.96 µm) during the whole span of the pulse, as proposed previously^8^. Yet, until real-time information of the magnetization dynamics and its interaction with the current pulse is available, this value remains uncertain.

For the three-segmented NW, the symmetric arrangement of the segments (Ni/Co/Ni) and the nature of the AMR measurement rendered the number of DWs and their positions too challenging to determine. Nonetheless, regardless of whether one or two DWs were actually pinned, the direction of the electron flow when current pulses were applied (i.e., the current pulse polarity) could always result in a de-pinning event. In the case of a single DW being pinned, it may have been pinned at one interface when one current polarity was applied, and then at the other interface when the reverse current polarity was used, resulting in a de-pinning event in both cases. This uncertainty in DW position can be evidenced in Supplementary Fig. S5b which shows that although in all experiments the pinning and de-pinning events occurred, not all de-pinning events happened at the same field, suggesting the magnetic configurations were not the same. In the case of two DWs being pinned, any polarity would result in de-pinning as there would always be electrons flowing from Ni into Co through the DW, as was found for the two-segmented. This is reflected in the current-induced switching at high enough pulse amplitudes and widths of both polarities: − 9.21 × 10^11^ A/m^2^ applied for 30 ns, + 1.38 × 10^12^ A/m^2^ applied for 20 ns and − 1.84 × 10^12^ A/m^2^ applied for 15 ns (see supporting figure S3).

The difference in magnitude of the peaks at around − 140 and − 240 Oe of “No pulse” from Fig. 4 suggests that the amount of magnetization that went from out of axis to aligned in the same direction of the applied measurement current was greater for the DW pinning than for the DW de-pinning. This could be explained by the vortices at the ends of the segments in state III Fig. 1 containing more magnetization out of axis than the pinned domain wall in state IV of the same figure, and as was previously found with micromagnetic simulations^11,18^. In addition to this, the peak around − 240 Oe for “9.21e11 A/m2” in Fig. 4, was higher in magnitude than the one shown for “No pulse” suggesting that the magnetization texture was modified either by the act of reducing the field to zero, the application of the current pulse, or both, while leaving the DW pinned.

From Fig. S6, using the linear approximation for resistor temperature dependence R = R_0_(1 + α ΔT), where R_0_ is the resistance at room temperature, α the temperature coefficient of resistance and ΔT the temperature increase from room temperature, it was possible to estimate the temperature of the NW system. A coefficient of α = 6 × 10^–3^ °C^−1^ for both Ni and Co^19^, led to a maximum temperature increase of 1.73 °C between the minimum and maximum resistances in Fig. S6. This resistive heating effect due to the continuously applied measurement current of 2 × 10^8^ A/m^2^ saturated as time passed. In addition to this, since no trend was observed for the de-pinning fields in Fig. 5 as the pulse width increased, the effect of current-pulse heating on DW de-pinning could be discarded. Furthermore, the presented method cannot resolve the effect of Oersted fields within the NW arising from the high current density pulses as positive and negative polarity de-pinning fields did not differ much from one another when both were observed.

Further experiments using the method described have to be performed on nanowires with different segment lengths and applying different current pulses and amplitudes to find the dependence of parameters like shape and crystal anisotropy on DW pinning and de-pinning. Also, the proposed method can only track the overall effect of the current pulse on one DW in two-segmented NWs, yet, it could be useful to quantify the effects of Oersted fields induced by the current pulses when coupled with magnetic imaging techniques^20^. This method is not limited to Ni/Co NWs and can be extended to any soft/hard magnetic materials combinations, length or diameter-modulated NWs, where a single DW can be pinned and the magnetization configurations are known. We believe this method will enable the measurement of current-DW interactions in such systems opening new possibilities for the study of prospective devices operating without an external magnetic field such as cylindrical NW logic and memory devices.

## Methods

### Alumina template preparation

Alumina membranes were prepared by hard anodization using 0.3 M oxalic acid (C_2_H_2_O_4_) in an electrolytic cell with a 99.999% electropolished aluminum disk as a working electrode and a platinum wired mesh as a counter electrode. For hard anodization, 40 V were first applied for 10 min and then ramped at 1 V/s to 140 V where it was sustained for 45 min while stirring and keeping the temperature around 0 °C. After this, the non-anodized aluminum was chemically etched using a 50:50 hydrochloric acid (HCl) aqueous solution with 1.7 g/100 ml Copper (II) chloride (CuCl_2_ 2H_2_O) addition. The alumina barrier layer was etched using 10 wt. % phosphoric (H_3_PO_4_) acid aqueous solution for 6:30 h to obtain a template open from both sides. On the chemical etched side, 100 nm of gold were sputtered to serve as working electrode for the subsequent electroplating.

### Cylindrical nanowires electroplating and isolation

Three different aqueous electroplating solutions were used. Gold: 1 g/L potassium dicyanoaurate (KAu(CN)_2_) and 40 g/L boric acid (H_3_BO_3_). Nickel: 300 g/L nickel sulfate hexahydrate (NiSO_4_·6H_2_O), 45 g/L nickel chloride hexahydrate (NiCl_2_·6H_2_O) and 45 g/L boric acid. Cobalt: 300 g/L cobalt sulfate heptahydrate (CoSO_4_·7H_2_O) and 40 g/L boric acid. Electroplating was performed galvanostatically using a Keithley 2400-C power source and controlling the deposited mass as the integral of the current over time, i.e., total deposited charge. The first three segments were electroplated in order (with current density in parenthesis): gold (− 0.5 mA/cm^2^), nickel (− 4 mA/cm^2^), cobalt (− 8 mA/cm^2^). After that, either nickel (− 4 mA/cm^2^) or gold (− 0.7 mA/cm^2^) were electroplated for 3 or 2 segmented wires, respectively. After deposition, the gold layer was plasma etched and the aluminum was chemically dissolved using an aqueous solution of 5 wt% phosphoric acid (H_3_PO_4_) and 1.8 wt% chromium trioxide (CrO_3_). The nanowires were then moved into ethanol by washing the chromium solution with fresh ethanol several times.

### Single nanowire electrical contact

2 µl of ethanol containing NWs were spread into 1 cm^2^ silicon substates with 150 nm thick silicon oxide surface and the ethanol let to evaporate. The sparse NWs were inspected using a focused ion beam capable scanning electron microscope and micrometer-sized marks were etched around a single wire to signal it. A two-step photoresist (LOR5B and AZ5214) photolithography process was used to create the four electrodes pattern using a Heidelberg µPG 501 and using the mentioned marks for alignment. After development, the samples were plasma etched in the load-lock after reaching a base pressure of 10^–7^ Torr and directly moved (without breaking vacuum) to a chamber where a 10 nm chromium and 180 nm gold were sputtered. Finally, the substrates were submerged in remover PG at 60 °C until lift-off was completed.

### Anisotropic magnetoresistance with current pulses

Substrates containing individual-contacted NWs were wire-bonded with aluminum to a sample holder which was floated by 10 kΩ from ground. All cables and connections were discharged and floated prior connecting the sample to the also floating sources and meters. The NW’s long axis was aligned parallel to the field generated by a pair of Helmholtz coils with iron cores, which were sourced using a KEPCO bipolar amplifier driven by a Keithley 2400-C. A Keithley 6221 current source and 2182A nanovoltmeter were used to perform a two-point-probe electrical measurement while the two remaining electrodes were contacted to an Agilent 33250A signal generator in charge of applying pulses. All instruments were controlled using LabView taking care that the signal generator and current source were not turned on at the same time, i.e., the current source was turned off when current pulses were applied and the signal generator was off while magnetoresistance measurements were being taken. A current density of 2 × 10^8^ A/m^2^ was used to measure AMR on all nanowires.

### Energy dispersive X-ray spectroscopy

Energy dispersive X-Ray spectroscopy was measured using a Nova Nano scanning electron microscope (SEM) equipped with an Octane elect plus detector. A line measurement was performed on the cross section of the membranes, along the long axis of the nanowires, using 15 kV acceleration voltage and 5.5 spot size (2.8 nA).

### AMR Micromagnetic simulation

Micromagnetic simulations of a Ni/Co NW of 600 nm segment length (1.2 um total) were performed using the Magpar^21^ package using an average edge length of 4.6 nm, an external field applied one degree away from the NW axis and material parameters tested before^11^. The magnetization was initialized with a complete saturation in the positive Z direction (m_z = 1) and allowed to relax. An external field was then applied in steps of 10 Oe from remanence up to − 500 Oe in the negative Z direction. The AMR values were extracted using Paraview “Calculator”, “Descriptive Statistics” and “Plot Data Over Time” filters.

## Supplementary Information

Supplementary Information 1.

Supplementary Information 2.

Supplementary Figures.

## Data Availability

The datasets used and/or analyzed during the current study available from the corresponding author on reasonable request.
