# Supplementary figures and images for: Current-induced zero-field domain wall depinning in cylindrical nanowires
Source: Sci Rep. 2022 Nov 14;12:19510. doi: 10.1038/s41598-022-22623-0 (PMC9663574; doi:10.1038/s41598-022-22623-0)

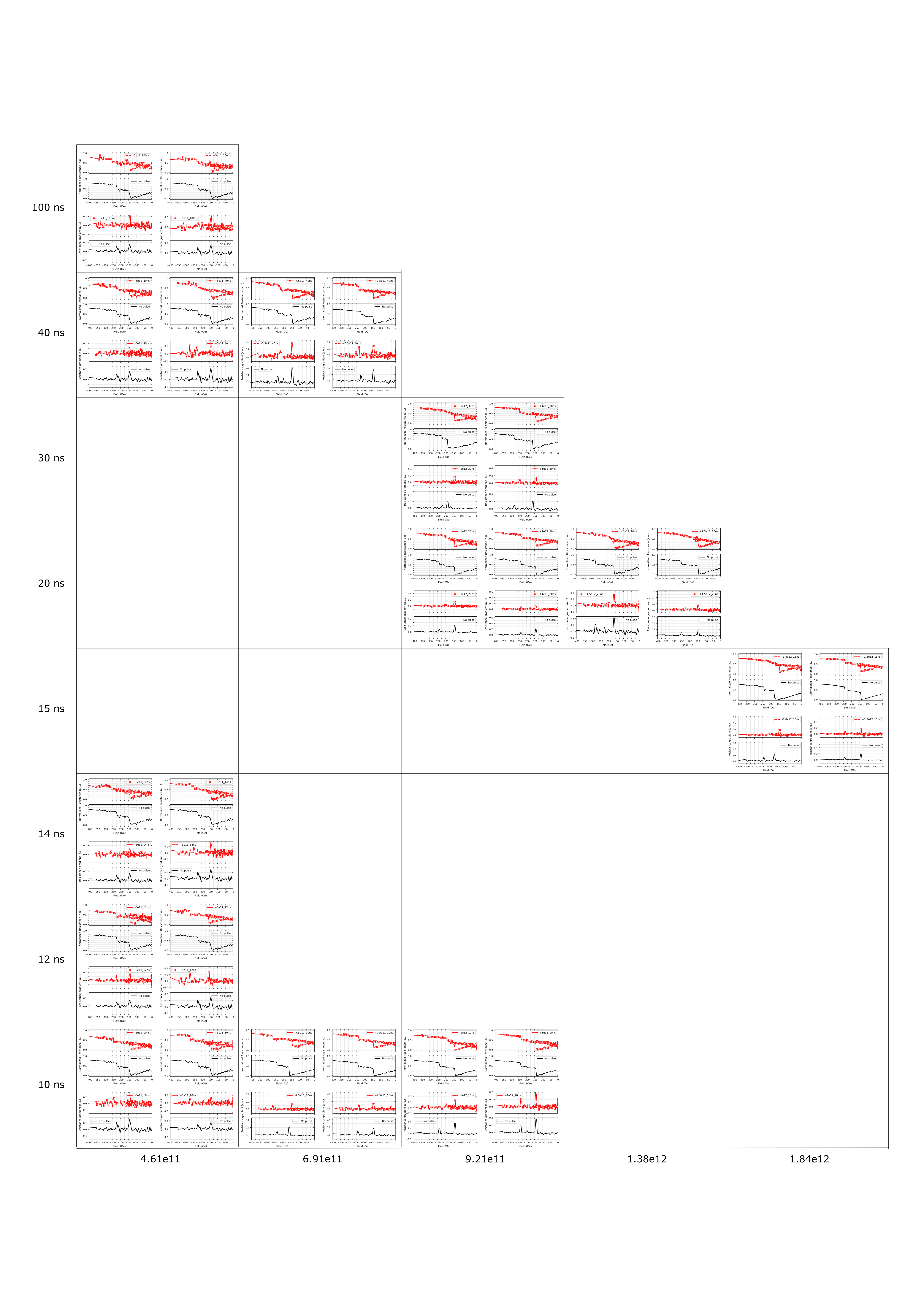

Supplement: Supplementary file 1 — Supplementary Information 1. [file 41598_2022_22623_MOESM1_ESM.png]

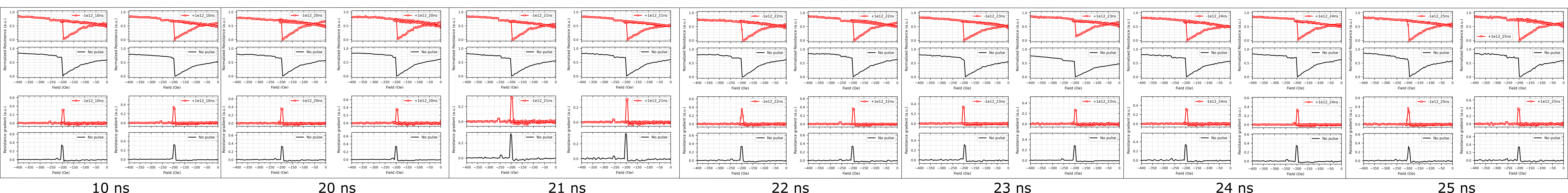

Supplement: Supplementary file 2 — Supplementary Information 2. [file 41598_2022_22623_MOESM2_ESM.png]
