# Supplementary material for: Current-induced zero-field domain wall depinning in cylindrical nanowires
Source: Sci Rep. 2022 Nov 14;12:19510. doi: 10.1038/s41598-022-22623-0 (PMC9663574; doi:10.1038/s41598-022-22623-0)
Supplement: Supplementary file 3 — Supplementary Figures. [file 41598_2022_22623_MOESM3_ESM.docx]

Supporting information: Current-Induced Zero-Field Domain Wall Depinning in Cylindrical Nanowires

Julian A. Moreno^1*^ and Jurgen Kosel^1, 2^

^1^ King Abdullah University of Science and Technology, Thuwal, Saudi Arabia, 23955

^2^ Silicon Austria Labs, Villach, Austria, 9524

* Correspondence and requests for materials should be addressed to J.M. (email: julian.moreno@kaust.edu.sa)


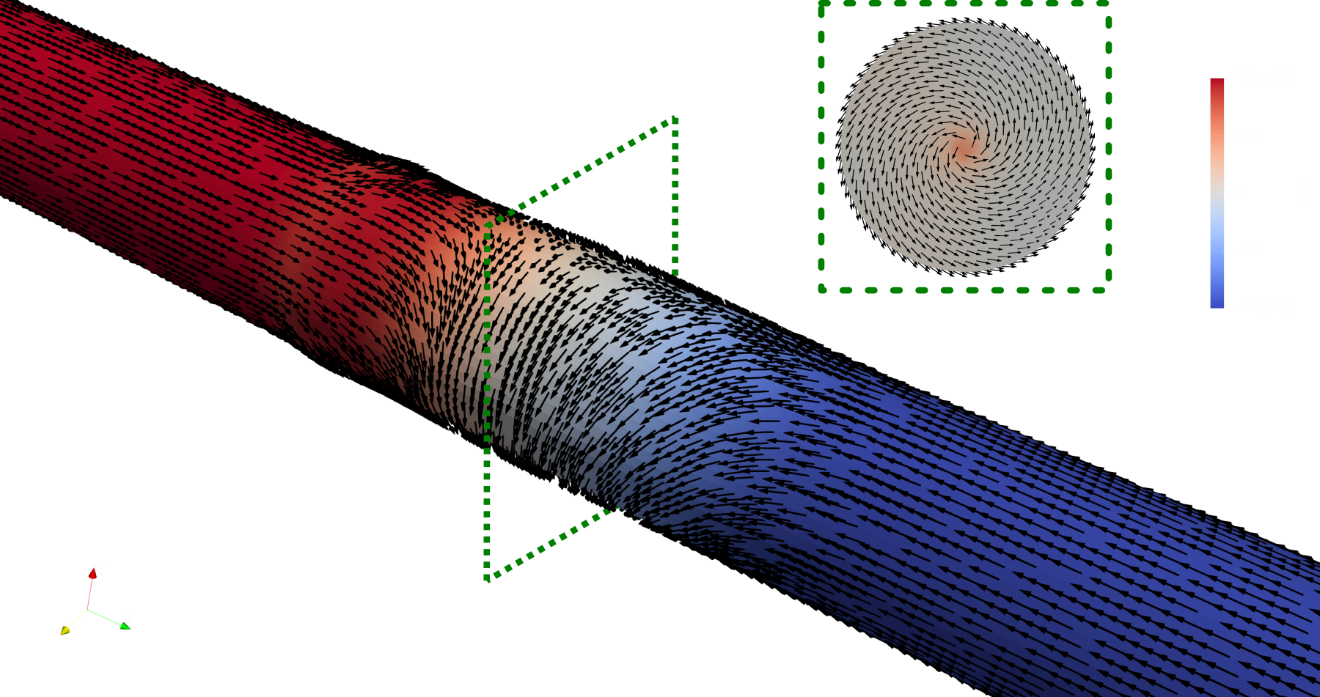


**Figure S1.** Micromagnetic structure of the domain wall from state IV of Figure 1b. The inset shows the cross section through the domain wall.


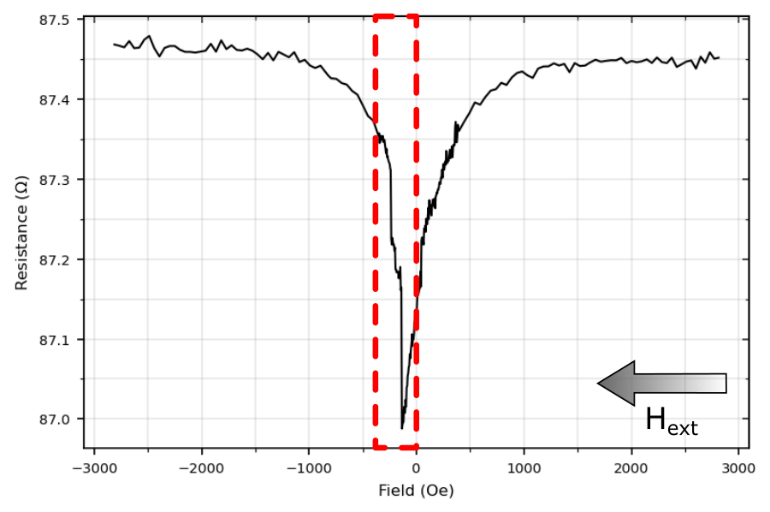


**Figure S2.** Typical full anisotropic magnetoresistance curve measured without sending a current pulse. The red, dashed box shows the selected relevant pinning/de-pinning range. The arrow indicates the direction of the applied field H_ext_: From 3kOe to - 3kOe.


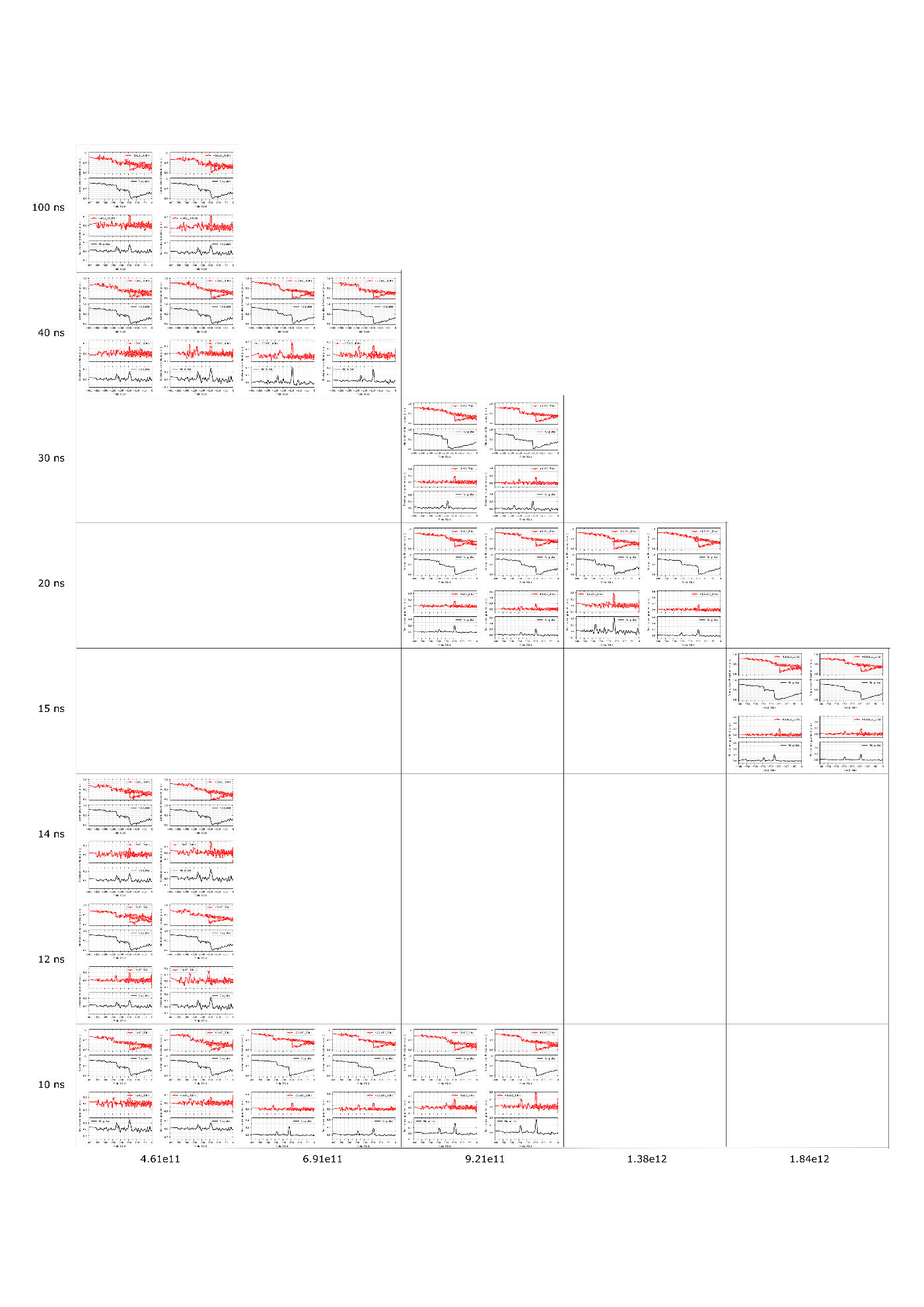


**Figure S3 (full resolution image included separately).** Array of plots of all measurements performed on the *three-segmented* nanowire. The x axis of the array shows the pulse amplitude in A/m2 and the y axis shows the pulse width. For each of these, four plots can be found: the top two show an AMR measurement where no pulse has been applied (black, solid line) and one after a pulse has been applied (red squares) for positive and negative polarities (see legends). The bottom two show the gradient of the top two figures.


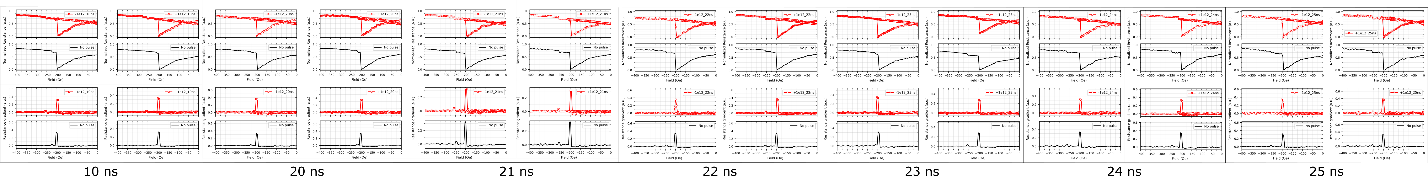


**Figure S4 (full resolution image included separately).** Array of plots of all measurements performed on the *two-segmented* nanowire. The x axis of the array shows the pulse widths used with a pulse amplitude of 1x10^12^ A/m^2^. For each of these, four plots can be found: the top two show an AMR measurement where no pulse has been applied (black, solid line) and one after a pulse has been applied (red squares) for positive and negative polarities (see legends). The bottom two show the gradient of the top two figures.

| a)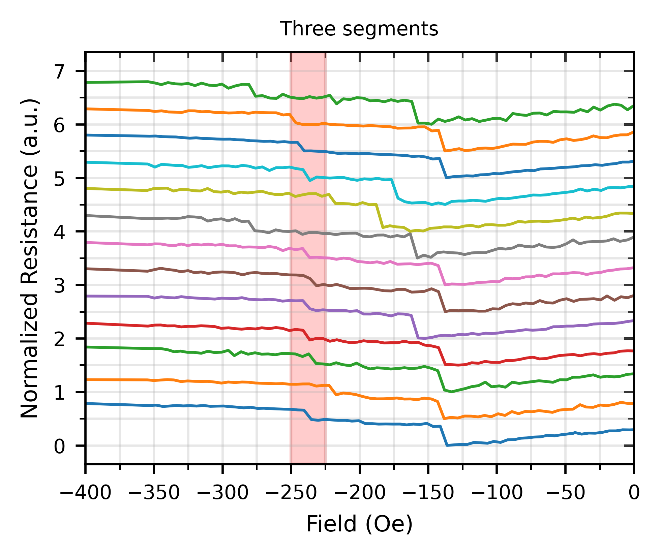 | b)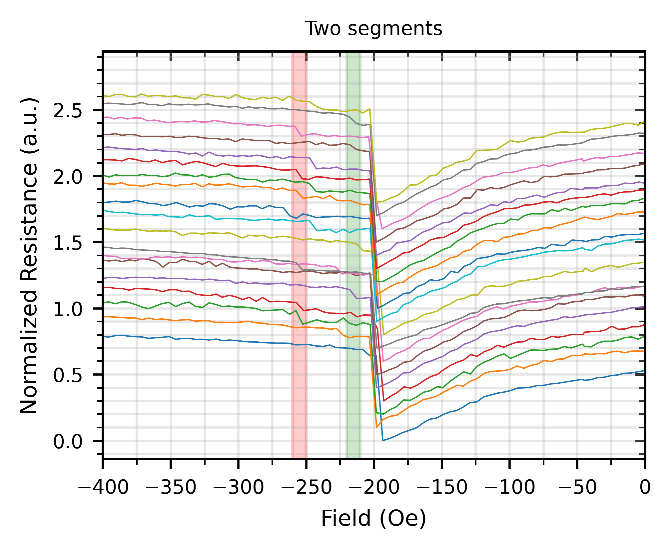 |
| --- | --- |

**Figure S5.** Measurements performed on three and two-segmented nanowires without applying current pulses. The red and green vertical regions indicate fields where most of the measurements presented a sharp resistance jumps, i.e., de-pinning fields.


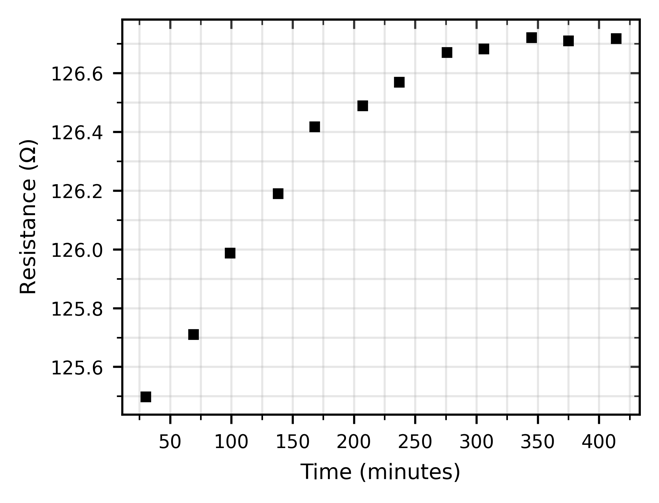


**Figure S6.** Minimum resistance extracted from the last day of AMR experiments.
